# CDK5/NFAT5-Regulated Transporters Involved in Osmoregulation in *Fejervarya cancrivora*

**DOI:** 10.3390/biology11060858

**Published:** 2022-06-03

**Authors:** Jiao Li, Xinru Wang, Tian Lan, Yingnan Lu, Meiling Hong, Li Ding, Lijun Wang

**Affiliations:** Key Laboratory for Ecology of Tropical Islands, College of Life Sciences, Hainan Normal University, Haikou 571158, China; 20202071300157@hainnu.edu.cn (J.L.); xinruwang@hainnu.edu.cn (X.W.); tianlan200830@hainnu.edu.cn (T.L.); ynlu192004@hainnu.edu.cn (Y.L.); mlhong@hainnu.edu.cn (M.H.)

**Keywords:** salinity, NKA activity, CDK5, transporters, ions

## Abstract

**Simple Summary:**

Amphibians mostly live in freshwater. However, crab-eating frogs (*Fejervarya cancrivora*) can adapt to high saline environments, while the osmoregulatory mechanism was not fully clear. Cyclin-dependent kinase 5 (CDK5), activating the nuclear factor of the activated T cells-5 (NFAT5) pathway, plays an important role in protecting cells from hypertonicity. We investigated the role of CDK5/NFAT5 in crab-eating frogs’ adaptation to saline environments. As a result, we found that the expression of CDK5/NFAT5 and its downregulated genes were higher in crab-eating frogs, which subsequently regulated ion transport to mediate osmotic pressure. The results indicate that CDK5/NFAT5-regulated transporters are involved in osmoregulation in frogs.

**Abstract:**

Crab-eating frogs (*Fejervarya cancrivora*) can live in brackish water with a salinity of up to 18‰, although most amphibians are not able to tolerate such high saline environments. To investigate its potential osmoregulation, we conducted experiments in *F. cancrivora* and *F. multistriata*. The results showed that *F. cancrivora* made use of ions (such as Na^+^ and Cl^−^) to increase intracellular concentrations via the Na^+^/K^+^-ATPase (NKA) enzyme. The mRNA expression of aldose reductase (AR) was significantly higher in *F. cancrivora* (*p* < 0.05), indicating that more organic osmolytes were produced and transported to maintain cellular homeosis. The mRNA expressions of Aquaporin 1 (AQP1) and AQP3 in kidney were significantly higher in *F. cancrivora*, while AQP expression in skin was higher in *F. multistriata* (*p* < 0.05). The mRNA level in activating the transcription of the nuclear factor of activated T cells-5 (NFAT5) which is one of the target genes of regulating the cellular response to hypertonicity, was higher in *F. cancrivora*. The protein expression of CDK5, the upstream protein of the NFAT5 pathway, was 2 times higher in *F. cancrivora.* Therefore, we can conclude that CDK5/NFAT5-regulated transporters might be involved in osmoregulation in *F. cancrivora*.

## 1. Introduction

Amphibians, depending on water, throughout their life stages are very sensitive to high saline conditions; therefore, they are at a high risk to severe environmental changes [1]. Some amphibians can adapt to high-saline water, for example, *Xenopus laeuis* can tolerate up to 600 m-osmoles; *Bufo viridis* can tolerate 800 m-osmoles; *Fejervarya cancrivora* can tolerate up to 1000 m-osmoles, with the high concentration comparable to seawater [2], while most frogs, such as rice frogs (*Fejervarya multistriata*), are not able to tolerate such high-saline environments. Crab-eating frogs (*Fejervarya cancrivora*), located around the coast of southeastern Asia, mainly inhabit bushes, mangrove swamps and forests [3]. Adult frogs can survive in 18‰ saline water, and their tadpoles can tolerate 35‰ marine water [4,5]. Studies have shown that the concentrations of sodium, chloride and potassium ions in blood plasma increased following a high saline trial in crab-eating frogs [4]. Nevertheless, the osmotic regulations of molecular mechanisms in crab-eating frogs are still largely unknown.

Some strategies of amphibian adaptive responses to hypertonicity have been researched [6], for example, *B. viridis* and *B. calamit* showed local adaptation to salinity [7,8]. There has been extensive focus on physiological traits, such as the concentration of permeable ions and ion transporters [2,9,10,11,12]. Na^+^, Cl^−^ and K^+^ are the three dominant ions responsible to homeosis in cells, and transporters play a crucial role in transporting these ions effectively. Na^+^/K^+^-ATPase (NKA), also known as sodium–potassium pump or sodium pump, is a key enzyme that controls extracellular volume via Na^+^ reabsorption, located on the membrane [13]. The NKA enzyme utilizes one molecule of ATP to export 3 Na^+^ and import 2 K^+^ through membranes in order to maintain intracellular homeostasis [14].

In addition, aldose reductase (AR) [15], the Na^+^/Cl_2_^−^ coupled betaine/c-aminobutyric acid transporter (BGT1) [16], the Na^+^/myo-inositol cotransporter (SMIT) [17], the Na^+^ and Cl_2_^−^ dependent taurine transporter (TauT) [18], and serum-and glucocorticoid-inducible kinase (SGK1) [19] also transport organic osmolytes to balance fluid volume. Cells require a large amount of osmolytes to elevate intracellular pressure. It was demonstrated that SGK1 regulates the transportation of Na^+^ and activates the enzyme activity of NKA in amphibians [20,21,22]. In kidney, aquaporins (AQPs) are responsible for water reabsorption via the renal tubules and collecting ducts from initial urine and therefore regulate urine concentration, which can be divided into 13 types in mammals [23,24,25]. It is reported that in amphibians, AQPs are generally classified into the following six types: 1, 2, 3, 5 and a1, a2. AQPs locate mainly on the urinary bladder and ventral pelvic skin to absorb water. For example, *Bufo japonicus* inhabits drier land, thus displaying several AQPs in order to transport water [26]. Urea transporter (UT-A) functions in urea accumulation, with four isoforms including UT-A1, UT-A2, UT-A3 and UT-A4. UT-A1 and UT-A2 can activate urea recycling and raise the concentration of urea while heat shock protein 70 (HSP70) can protect cells from high urea [27,28,29,30]. These proteins work together in the system to prevent damage from hypertonicity (see review [31]).

When cells encounter hypertonic conditions, cyclin-dependent kinase 5 (CDK5) can be activated, following which CDK5 regulates the nuclear factor of activated T cells-5 (NFAT5), also named TonEBP, which primarily functions in regulating the cellular response to hypertonicity [32]. Renal medulla metabolizes salt and urea. When renal medulla occurs under hypertonic conditions, it causes double-stranded DNA breaks [33]. CDK5/NFAT5 can up-regulate the expression of osmoprotective genes, including AR, BGT1, SMIT, TauT, SGK1, AQPs, UT-A and HSP70, which could increase the intracellular concentration, and thus protect cells from DNA damage [23]. Therefore, CDK5/NFAT5 pathway plays an essential role in the regulation of maintaining the fluid volume to protect cells from hypertonicity.

The aim of this study was to investigate possible molecular response to high saline environments in *F. cancrivora*, via comparison with *F. multistriata*. We measured the concentration of ions and NKA enzyme activity to compare two frogs’ responses on the physiological level, followed by the detection of the expression of CDK5/NFAT5 and its downregulated genes.

## 2. Materials and Methods

### 2.1. Animal Sampling

*F. cancrivora* were collected in June 2021 from Dongzhai Port Nature Reserve, China (110°34′31.72 E, 19°57′2.81 N) and *F. multistriata* were collected in farms near the Dongzhai Port. Six adult crab-eating frogs were caught and placed in boxes with natural salt water in mangrove, and the average salinity of the mangrove water was 14‰ (Supplementary Table S1), while six adult *F. multistriata* were placed in another box with freshwater, followed by transference to the laboratory immediately to measure their body mass (±0.1 g; BW) and snout-vent length (±1 mm; SVL) (Supplementary Table S2). Frogs were euthanized with ethyl carbamate after measurements. Cardiocentesis for blood collection was performed on frogs with 1 mL clean medical syringes and needles injected in the heart. The blood (200 μL) of each frog was collected in tubes with heparin anticoagulant for biochemical analysis. Then, dissection was performed on frogs and skin, liver and kidney tissues were collected for RNA extraction.

### 2.2. Biochemical Analysis

Blood samples were immediately centrifugated at 3000 rpm for 5 min. Plasma was pipetted into a fresh 1.5 mL microfuge tube after centrifugation. Concentrations of Na^+^, Cl^−^, K^+^ were detected using the ISE internal standard (HITACHI, Tokyo, Japan) [34]. ISE is a method of using ion-selective electrodes to detect concentrations of ions from samples. The electromotive force produced by ion concentrations and sensitivities from all samples is calculated with Nernst formula:

(1) $$E=E_{0}+\frac{2.303\;R\;T}{n\;F}loga_{x},$$

(2) $$a_{x}=f_{x}\times C,$$

where *E* is the electromotive force in the measured samples, *E*_0_ is the standard electromotive force in the measurement systems, *n* is the charge number of ions to be tested, *R* is the gas constant (8.314 J/K mol), *T* is the absolute temperature (273 + t °C), *F* is the Faraday constant (96,487 C/mol), *a_x_* is the ionic activity to be tested, *C* is the concentrations of ions to be tested, and *f_x_* is the ionic activity coefficient to be tested.

### 2.3. NKA Enzyme Measurement

Samples were homogenized 1:10 in normal saline, then centrifuged at 4000 rpm at 4 °C for 10 min after which the supernatant was collected for determining protein concentrations using the BCA method with an assay kit (Nanjing Jiancheng, Ltd., Nanjing, China). The enzyme activity of NKA was detected following the manufacturer’s instructions (Nanjing Jiancheng, Ltd., Nanjing, China). Samples were mixed with the matrix solution, ddH_2_O as control, followed by reactions at 37 °C for 10 min. Chromogenic reagents were added to the control and NKA groups, then samples were centrifugated at 3500 rpm for 10 min. Supernatant was collected for the phosphate assay. The OD (optical density) value was detected at 636 nm using an enzyme-labeled instrument (BIO-RAD, Hercules, CA, USA) to calculate the enzyme activity of NKA.

### 2.4. Total RNA Extraction and cDNA Synthesis

Total RNA was extracted from tissues according to the manufacturer’s instructions for the Trizol reagent (Invitrogen, Carlsbad, CA, USA). Briefly, 100 mg tissue samples were added to 1 mL of the Trizol reagent, and were later smashed using a Tissue Grinder (Scientz, Ningbo, China). The supernatants were collected after adding chloroform for the purpose of final precipitation with the same volume of isopropanol. The precipitation was washed twice with 75% ethanol and eventually resuspended in 30 μL DEPC-treated water. The concentration and quality of total RNA was measured by NanoDrop one/onec spectrophotometer (Thermo Fisher, Waltham, MA, USA). The integrity of RNA was detected using 1.2% agarose gel electrophoresis. Qualified RNA then was reverted into cDNA using the cDNA synthesis kit (Invitrogen, Carlsbad, CA, USA).

### 2.5. Quantitative Real-Time PCR (qRT-PCR)

All primers were designed according to the whole transcriptome sequencing data (Supplementary Tables S3 and S4). The nucleotide sequences of primers are provided in Table 1. β-tubulin, compared with β-actin and GAPDH, was selected as the reference gene for steadiness. A qRT-PCR was performed with the Genius 2 × SYBR Green Fast qPCR MixSKit (ABclonal Technology, Boston, NY, USA) to detect the expression of selected genes. The total volume of the reaction was 20 μL, including 1 μL cDNA sample, 10 μL 2 × Mix, 0.8 μL each of the 10 mM forward and reverse primers and 7.4 μL of dH_2_O. The 2^−ΔΔCt^ method of processing data was used to obtain the relative expression levels of mRNA. The values of skin in *F. multistriata* were standardized to 1. 

### 2.6. Western Blotting

Tissues were cut into pieces and lysed using an RIPA lysis buffer with 3 μL nuclease and 5 μL PMSF separately, then later smashed using a Tissue Grinder (Scientz, Ningbo, China). The supernatants were collected and denatured at 37 °C for 30 min. Subsequently, the concentrations of protein were measured using a BCA assay (Nanjing, Jiancheng). After the application of the BCA assay, all samples were adjusted to the same concentration with a loading buffer and boiled for 6 min, then proteins from *F. cancrivora* and *F. multistriata* were mixed, respectively, and stored in a refrigerator. All equivalent amounts of protein samples were separated by 8–10% sodium dodecyl sulfate-polyacrylamide gel electrophoresis (SDS-PAGE) followed by transference to polyvinylidene difluoride (PVDF) membranes (Millipore Corp, Atlanta, GA, USA). The membranes were placed in 3% nonfat dried milk at room temperature for 2 h and washed with TBST 3 times and immunoblotted with specific primary antibodies (Supplementary Table S5), followed by incubating at 4 °C overnight. HRP-conjugated secondary antibodies (BOSTER Biological Technology, Wuhan, China) were incubated for another 1 h and washed with TBST 4 times. The signal was detected using Pierce ECL reagent after washing (Thermo Fisher Scientific, Waltham, MAUSA) with LSA 4000 mini ImageQuant instrument (CE Healthcare, Chicago, IL, USA).

### 2.7. Statistical Analysis

All data were represented as Mean ± S.E. analyzed using the SPSS 16.0 software (IBM SPSS, Chicago, IL, USA). An independent-sample T test was used to test the significance of the ion concentration and activity of NKA between the two species. One-sample Kolmogorov–Smirnov and Homogeneity of variance tests were carried out to evaluate the normal distribution and statistical homogeneity of data. The significance of the relative expressions of selected genes and proteins between frogs was determined using an independent-sample T test or Mann–Whitney U test. Statistical significance was set at *p* < 0.05. In order to quantify the results of the Western blot analysis, a gray level test was used to calculate the protein expression level of CDK5. ImageJ was utilized to calculate the gray level of proteins. All figures are presented via Graphpad prism 7.0 (GraphPad Software, San Diego, CA, USA). 

## 3. Results

### 3.1. Ion Concentration in Plasma

**Figure 1:**
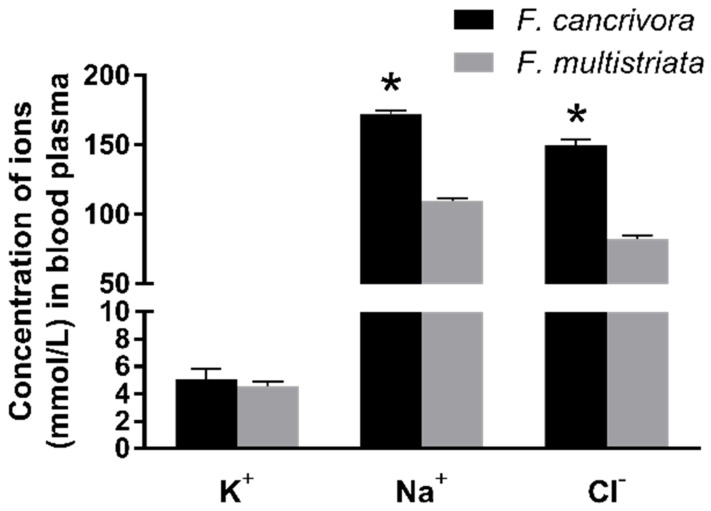
Concentrations of Na^+^, Cl^−^, K^+^ in *F. cancrivora* and *F. multistriata* in blood plasma. * Statistical significance when *p* < 0.05 in independent-sample *t*-test.

The mean concentration of K^+^ in *F. cancrivora* was 5.03 mmol/L and *F. multistriata* was 4.54 mmol/L; however, there was no significant difference between these two species (*p* > 0.05). Nonetheless, Na^+^ and Cl^−^, the two main ions in seawater, were significantly different in the two species. The mean concentration of Na^+^ in *F. cancrivora* was 171.73 mmol/L, higher than 109.34 mmol/L in *F. multistriata* (*p* < 0.01), as the mean concentrations of Cl^−^ in two species were 149.4 mmol/L and 82.4 mmol/L, respectively (*p* < 0.05) (Figure 1, Supplementary Table S6).

### 3.2. NKA Enzyme Activity

**Figure 2:**
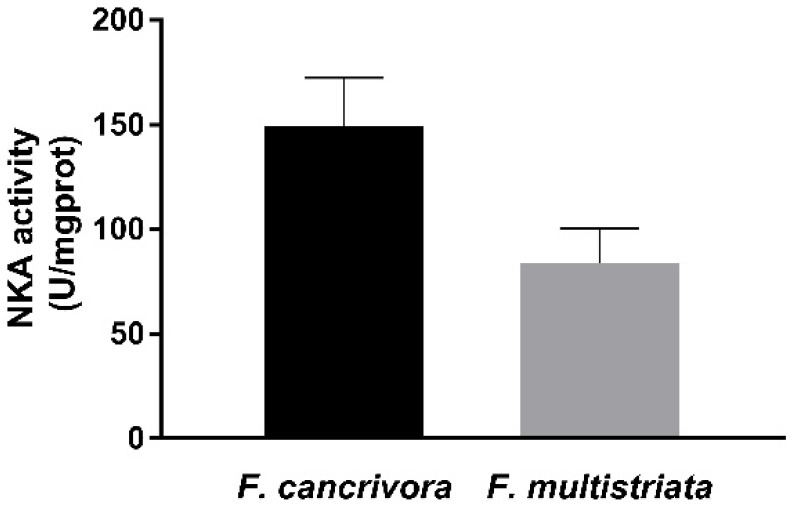
Activity of NKA enzyme in *F. cancrivora* and *F. multistriata* in liver. Independent-sample T test was used to calculate significance.

The activity of NKA was detected in *F. cancrivora* as 149.36 ± 23.11 U/mgprot, while in *F. multistriata* the activity was 83.67 ± 16.7 U/mgprot. The activity of *F. cancrivora* was 1.8 times higher, but with no significant difference (*p* > 0.05) (Figure 2).

### 3.3. The mRNA Expression of Osmoregulative Genes

The expressions of osmoregulative genes overall were highest in kidney and the expression level in skin and liver was almost identical in the two species. 

In kidney, AR was significantly expressed to a higher degree in *F. cancrivora* (*p* < 0.05), while SGK1, SMIT, TauT were slightly higher (*p* > 0.05). However, the expression of BGT1 was a little higher in *F. multistriata* (*p* > 0.05). It showed no significant difference between these genes in skin and liver (*p* > 0.05). 

In kidney, the mRNA expressions of AQP1, AQP3 were significantly higher in *F. cancrivora* (*p* < 0.05), increasing by 2.3–2.5 fold, but no significant difference was detected in AQP4 (*p* > 0.05). In skin, the expressions of AQPs were higher in *F. multistriata* with a significant expression of AQP4 (*p* < 0.05). In liver, there was no significant expression variance in the AQPs between two species (*p* > 0.05). 

**Figure 3:**
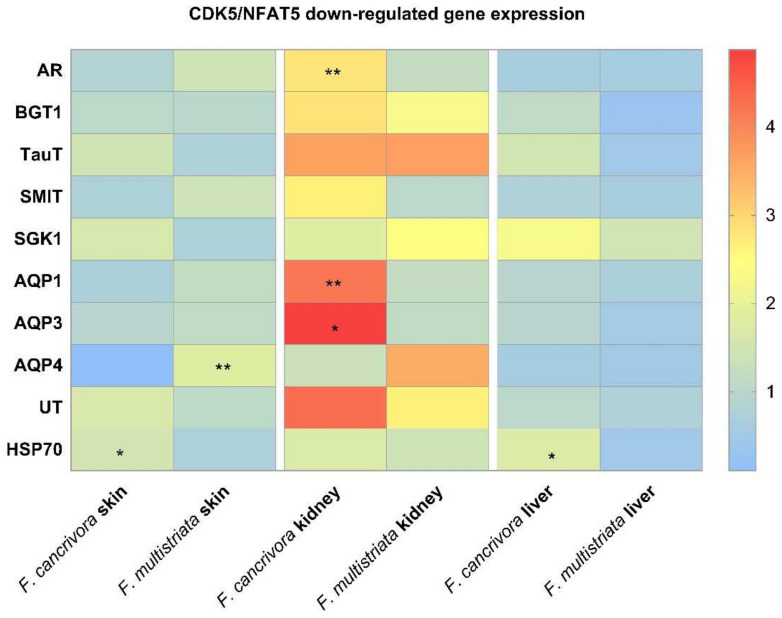
The mRNA expression of transporters: *F. cancrivora* skin standardized to 1, * Statistical significance when *p* < 0.05, ** when *p* < 0.01.

No significant difference in the expression of UT-A appeared in all tissues (*p* > 0.05), but in kidney, *F. cancrivora* increased by 1.6 fold. In skin and liver, UT-A also presented a slightly higher expression in *F. cancrivora*. As for HSP70, it was significantly expressed at a higher degree in skin and liver tissues in *F. cancrivora* (*p* < 0.05). (Figure 3).

### 3.4. The Expression of NFAT5 and CDK5

**Figure 4:**
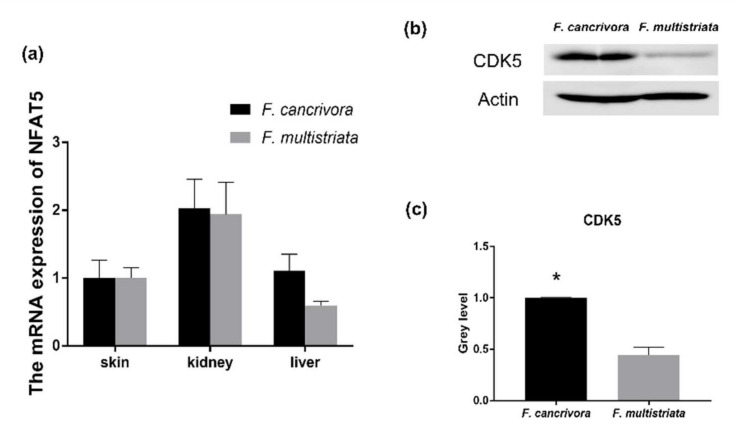
The mRNA expression of NFAT5 and the protein expression of CDK5 in kidney. (**a**) The mRNA expression of NFAT5: *F. cancrivora* skin standardized to 1; (**b**) the representative bands of CDK5 protein expression; (**c**) gray analysis of CDK5: *F. cancrivora* standardized to 1. * Statistical significance.

The mRNA expression of NFAT5 was highest in kidney, and NFAT5 was also expressed in skin and liver tissues. *F. cancrivora* demonstrated a slightly higher expression level in skin, kidney and liver, but with no significance (*p* > 0.05). We calculated the quantity of the protein expression of CDK5 using gray level (Supplementary Table S7, Figure S1) and found that *F. cancrivora* was 2 times higher (Figure 4).

## 4. Discussion

Ions play an important role in regulating cell conditions when organisms are transferred from seawater to freshwater to adapt to environments which has been reported in fishes [35], crustacea [36], and frogs [37]. For example, *Liza haematocheila* juveniles tend to raise concentrations of K^+^, Na^+^ and Cl^−^ in serum with elevating salinity [38], as do *Acipenser schrenckiiin*, leading to increased concentrations of Na^+^ and Cl^−^ in serum while no significant difference of K^+^ is observable [39]. In the present study, concentrations of Na^+^ and Cl^−^ in the plasma of *F. cancrivora* were much higher than *F. multistriata*. Frogs with permeable skin could allow ions to alter higher external salinity to promote lower internal concentrations. Hence, the progressive increment of these ions in *F. cancrivora* might be due to permeation, which was also reported in *Rana pipiens* after they were exposed to seawater [2]. Nevertheless, the concentration of K^+^ was 3.6~5.6 mmol/L in two species and presented no significant difference, consistent with *Acipenser schrenckiiin*’s state, which can be attributed to the preservation of the integrity of cell membranes [39,40].

Na^+^/K^+^-ATPase (NKA) is a crucial enzyme in ion transport, where Na^+^ could permeate from intracellular to extracellular fluids while K^+^ does the opposite in order to reach osmotic equilibrium [13]. It was reported that *Bufo balearicus*, which also lives in brackish water, presented much higher activity of the NKA enzyme than *Bufo bufo*. Furthermore, the activity increased in response to seawater exposure [12]. Juvenile bull sharks (*Carcharhinus leucas*) showed an elevated enzyme activity when transferred from freshwater to seawater [41]. In this study, the activity of the NKA enzyme in *F. cancrivora* was higher according to the ion concentration results, implying that NKA is an important ion transporter for *F. cancrivora* to ensure sufficient ions are provided quickly to maintain cellular homeostasis, and adapt to the brackish environment, similarly to teleost fish [42,43].

**Figure 5:**
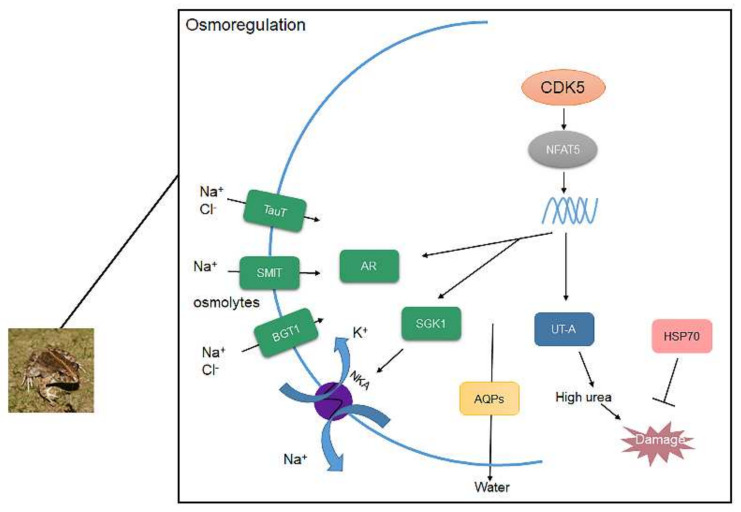
The conclusion of CDK5/NFAT5 mediating downstream genes to response to high saline environments.

To maintain cellular homeostasis, organic osmolytes are also important to increase fluid volume. AR is the key enzyme of the glucose metabolism sorbitol pathway, which can transform glucose into sorbitol, an organic osmolyte [44,45], thereby increasing concentrations in cells. According to our study, the mRNA expression of AR was expressed at a significantly higher rate in *F. cancrivora*. In rats, hypertonicity induces the mRNA expression of AR [46] as AR is an osmoprotective gene. Inositol and taurine are synthesized in renal cells and can be transported by SMIT and TauT [47,48]. Under hypertonic conditions, inositol and taurine are transported into cells rather than synthesized in vivo; therefore, hypertonicity activates the mRNA expression of inositol and taurine transporters [18,49,50,51]. In this study, SMIT and TauT were expressed at a slightly higher rate in *F. cancrivora*. We assumed that frogs have a different osmoregulation mechanism in that they mainly utilize AR to produce osmolytes to maintain higher concentration in cells rather than transport osmolytes; therefore, SMIT and TauT could be “backup genes” in *F. cancrivora*. Besides, SMIT and TauT can also transport Na^+^ and Cl^−^ into cells. It is possible that these transporters could co-transport ions with NKA. Betaine is synthesized from choline both in liver and kidney [47]. BGT1 transports betaine into cells and hypertonicity stimulates its expression in rats [50]. However, our results showed that BGT1 was expressed at a higher rate in *F. multistriata* in kidney. BGT1 is not only regulated by transcription but is also related to plasma membrane insertion [52]. Therefore, the result could be altered depending on the effects of multiple factors under this specific circumstance. Moreover, SGK1 mediates the aldosterone regulation of Na^+^ transport [20,21], resulting in the activation of NKA in amphibian renal epithelial cells [22]. Based on our results, the expression of SGK1 was higher in *F. cancrivora*, which is in accordance with the results of the enzyme activity of NKA. Therefore, Na^+^, Cl^−^, and osmolytes can be transported into cells via these transporters and NKA, causing an increase in the intracellular concentration (Figure 5).

It is widely reported that NFAT5 can activate transporters and AR to regulate organic osmolytes permeating through cell membranes [53,54]. NFAT5 was originally identified as a key transcription factor in the kidney medulla under hypertonic conditions [23,32,55]. Cyclin-dependent kinase 5 (CDK5) is one of the activators of NFAT5. In this study, the protein expression of CDK5 was 2 times higher in *F. cancrivora*; however, the transcriptional expression of NFAT5 was not significantly different, as it was slightly higher in *F. cancrivora* in three different tissues. This suggests that up-stream protein CDK5 might activate NFAT5, and that NFAT5 relies on its transcriptional activation activity to regulate its target genes and stimulate the production of transporters. Therefore, under high saline environments, in order to adapt to environmental conditions and regulate hypertonic cellular conditions, CDK5/NFAT might be triggered in *F. cancrivora* (Figure 5).

CDK5/NFAT5 also stimulates aquaporins (AQPs) and the urea transporter (UT-A) to produce the amount of urea in kidney and HSP70, which is often overexpressed to protect cells from high urea [31]. AQPs is a complex family with 13 different types. AQP1 is highly expressed in kidney to control water reabsorption. AQP3 is more efficient in transporting glycerol. AQP4 amplifies the function of urea concentration through AQP3 [25]. In kidney, the expression of AQPs was significantly higher in *F. cancrivora*, while in skin, *F. multistriata* had a much higher trend. It is believed that expressing more AQP1 is beneficial for excluding water from kidneys and AQP3 could transport small molecules such as urea into cells in *F. cancrivora*, thereby increasing the concentration of urea to maintain a hyperosmotic cell pressure. Therefore, a high expression of AQPs is necessary in kidney, and a lower expression of AQPs in skin prevents environmental water from entering the body. *Takifugu obscurus* also presented the same results when subjected to salinity change [56] (Figure 5).

High urea is crucial to frogs to elevate concentrations in the body [2,57]. UT-A can carry a large number of small urea molecules quickly through membranes, thereby maintaining an extremely high osmotic pressure and concentrated urine [58]. However, high urea can be damaging to health; to protect cells, organisms should develop an appropriate response mechanism. Moreover, hypertonicity can cause protein oxidation in cells [23]. Liver, as the main metabolic organ, plays a key role in producing protective proteins such as HSP70. For instance, HSP70 is overexpressed to inhibit apoptosis, induced by various stressors [59], or to protect cells from high urea. It was reported in rats that HSP70 was expressed under high salinity for cell survival [60]. In this study, UT-A and HSP70 were also highly expressed in *F. cancrivora*, indicating that urea plays a crucial role in osmoregulation, and HSP70 can protect against high urea for *F. cancrivora* (Figure 5).

Various environmental factors affect physiological indicators; however, we found that the salinity of the two species’ habitats differs greatly. The average salinity of mangrove water was 14‰ based on our detection and that of fresh water was less than 0.05‰. Therefore, salinity could be a very important factor that affects physiological mechanisms. It is well established that the environment that individuals inhabit can strongly affect physiology as an adult. Therefore, any differences observed may be simply from occupying different environments, not due to species-specific differences in physiology. For example, *F. cancrivora*, *Bufo viridis* and *Xenopua laeuis* had increased ion concentrations when transported into high saline environments. Hence, environments affect physiological mechanisms in species. However, these frogs still displayed much higher ion concentrations in freshwater compared with other species in the same taxa [2]. This indicates that the species is capable of adapting to high salinities, and the levels of plasma sodium can be very high, implying considerable tolerance to high plasma sodium in these species, which can also be seen in our results. This could provide evidence of adaptive responses to high saline environment in *F. cancrivora*. When comparing the osmoregulatory systems of two closely related kajika frogs, *Buergeria japonica* (brackish water living) and *B. buergeri* (freshwater living), the plasma Na^+^ concentration was also significantly higher in *B. japonica* than in *B. buergeri*, suggesting differences in the mechanisms for salinity adaptation [61], which is similar to our results. Therefore, the differences among our results in the two species could be attributed to salinity adaptation.

## 5. Conclusions

In conclusion, *F. cancrivora* relies on Na^+^ and Cl^−^ transport through NKA and perhaps SMIT, TauT and BGT1 co-transport ions with NKA. AR and AQPs in kidney were highly expressed in *F. cancrivora*, which indicated that more organic osmolytes were produced and more water was filtered to maintain cellular homeosis. CDK5, as the upstream protein, was highly expressed in *F. cancrivora* and activated the transcription of NFAT5, which primarily plays a role in regulating cellular responses to hypertonicity. Hence, CDK5 activates NFAT5, and NFAT5 therefore regulates these target transporters to protect cells from hypertonicity. *F. cancrivora* could rely on these transporters to survive in high saline environments (Figure 5).

## Figures and Tables

**Table 1:** Sequences of primer pairs for qRT-PCR in *Fejervarya cancrivora* and *Fejervarya multistriata*.

Gene	*Fejervarya cancrivora*	*Fejervarya multistriata*
NFAT5	CGGTCCTTAACATTTCGCCA CACCCCCTTCATCTTGGCAT	CGGTCCTTAACATTTCGCCA CACCCCCTTCATCTTGGCAT
AQP1	GTACATCATCGCCCAGTGCC CAAGGCCAGAGGTGATACCG	GTACATCATCGCCCAGTGCC CAAGGCCAGAGGTGATACCG
AQP3	TGGGATCTTCGCCACCTTTC AGCGGTGCCAATAAACTGGT	TGGGATCTTCGCCACCTTTC AGCGGTGCCAATAAACTGGT
AQP4	CTGATGTTGCTGGAGGTTTGG TACCAGAAGACCGTGACCAG	CTGATGTTGCTGGAGGTTTGG TACCAGAAGACCGTGACCAG
BGT1	CAGGACCGGGATTGGCTTT GAAGAAGAGGCAGGACCAGA	GTTACAGGACCGGGATTGGC GAAGAAGAGGCAGGACCAGAG
SMIT	TGGGTCAAACTCCAGCTTCT TTGCTGCCAAAACCCTTTGA	GCTGTGATGATTGCGGCTCT CAAGAGTGAAGATGGTGCTTGC
TAUT	TATTTCACGGCCACCTTCCC CAGTCGTGTGATGTTCGGGT	TATTTCACGGCCACCTTCCC CAGTCGTGTGATGTTCGGGT
SGK1	GCCAAGCCATCAGACTTCCA ATCAGCTTTGTGTCGTGCCA	GCCAAGCCATCAGACTTCCA ATCAGCTTTGTGTCGTGCCA
HSP70	GATGCCACACAAATCGCTGG CGAACACAACAATCCTCGGC	ATCGCTGGACTCAACTGTCT TGAATGTCCCATGTCCACGA
AR	GCTGCCAACCAATCTTCC ATGCCTTTGCCCACTTCA	GCTGCCAACCAATCTTCC ATGCCTTTGCCCACTTCA
UT-A	GCGTGGAGCATCTCAAGTCA GAGCCACCCATACCAGTCAC	GCGTGGAGCATCTCAAGTCA GAGCCACCCATACCAGTCAC
β-tubulin	CTGGCTGTCAACATGGTCCC TACTGTTGGCTACCACGGCT	

## Data Availability

All data relevant to this study are provided in the manuscript and Supplementary Materials.

## Supplementary Materials

The following supporting information can be downloaded at: https://www.mdpi.com/article/10.3390/biology11060858/s1, Figure S1: Original images of Full Western Blot, Table S1: The salinity of mangrove water; Table S2: Morphometrical parameters of *Fejervarya cancrivora* and *Fejervarya multistriata*; Table S3: KEGG pathway of unigenes *Fejervarya cancrivora*. Table S4: GO enrichment of unigene in *Fejervarya cancrivora*; Table S5: Primary antibodies of Western blotting; Table S6: Concentration of ions in blood plasma in *Fejervarya cancrivora* and *Fejervarya multistriata*; Table S7: Gray level of CDK5 and β-actin in *Fejervarya cancrivora* and *Fejervarya multistriata*.
