# Supplementary material for: CDK5/NFAT5-Regulated Transporters Involved in Osmoregulation in Fejervarya cancrivora
Source: Biology (Basel). 2022 Jun 3;11(6):858. doi: 10.3390/biology11060858 (PMC9220195; doi:10.3390/biology11060858)
Supplement: Supplementary file 1 [file biology-11-00858-s001.zip › Supplementary materials.pdf]

Supplementary Table S1. The salinity of mangrove water.

Time	Salinity of duck	Salinity of center of mangrove
2020/12/27	17‰	12‰
9:45	17‰	11‰
	15‰	11‰
	13‰	11‰
	13‰	11‰
12:09	9‰	10‰
	10‰	10‰
	10‰	10‰
	10‰	10‰
	9‰	10‰
15:18	6‰	6‰
	6‰	6‰
	6‰	6‰
	6‰	7‰
	6‰	8‰
17:35	10‰	11‰
	10‰	11‰
	10‰	11‰
	10‰	11‰
	10‰	12‰
2020/12/28	14‰	
2021/04/05	17‰	
2021/06/02	15‰	

Supplementary Table S2. Morphometrical parameters of *Fejervarya cancrivora* and *Fejervarya multistriata*.

F. cancrivora	BW (g)	SVL (mm)
1	29.9	70.35
2	32	72.22
3	56	81.3
4	9	45.6
5	19	61.86
6	11	53.5
F. multistriata		
1	16	55.16
2	16	57.22
3	20	52.75
4	15	54.98
5	17	59.83
6	21	62.87

Supplementary Table S3. KEGG pathway of unigenes *Fejervarya cancrivora* for primer use.

Supplementary Table S4. GO enrichment of unigene in *Fejervarya cancrivora* for primer use.

Supplementary Table S5. Primary antibodies of western blotting.

Gene	Host	Dilution	Manufacturer
CDK5	Rabbit	1:500	ABclonal Technology Boston, USA
β-actin	Rabbit	1:10000	ABclonal Technology Boston, USA

Supplementary Table S6. Concentration of ions in blood plasma in *Fejervarya cancrivora* and *Fejervarya multistriata*.

Species	Ions	Mean ± S.E.
F. cancrivora	K^+	5.03 ± 0.8
	Na^+	171.73 ± 2.6
	Cl^-	149.4 ± 4.2
F. multistriata	K^+	4.54 ± 0.3
	Na^+	109.34 ± 2.2
	Cl^-	82.4 ± 2.2

Supplementary Table S7. Grey level of CDK5 and β-actin in *Fejervarya cancrivora* and *Fejervarya multistriata*.

Species	Integrated density of CDK5	Integrated density of β-actin
F. cancrivora	213802	481199
	588670	1278255
	610792	1355019
F. multistriata	23033	407153
	271022	924957
	263733	1032992

(1) Actin 1

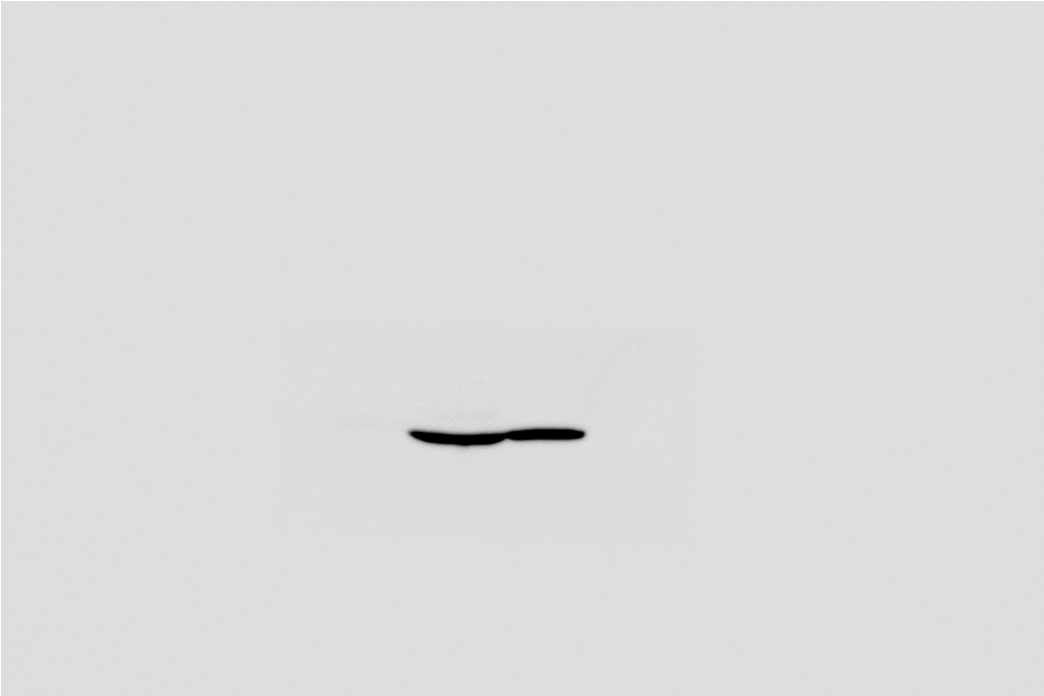

(2) Actin 2

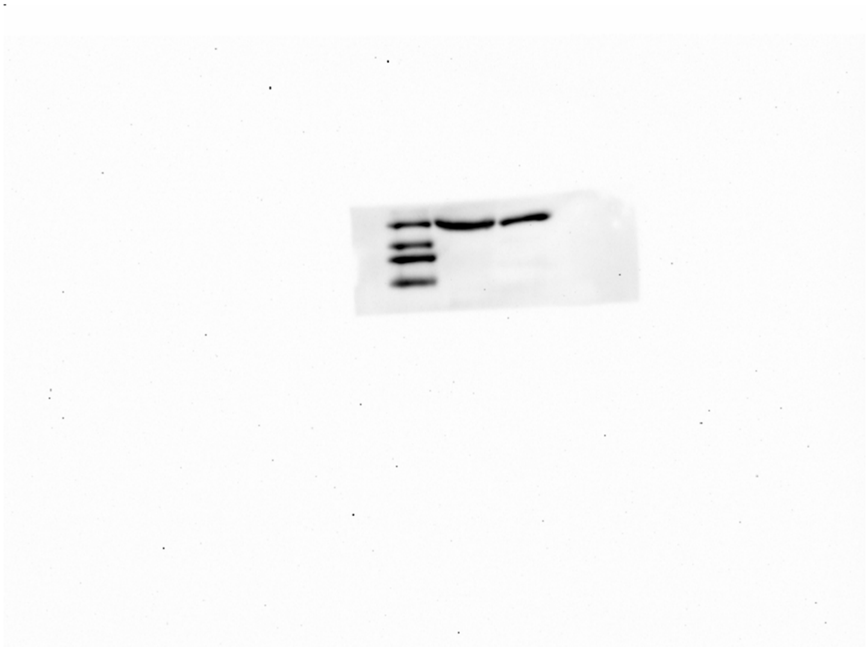

(3) Actin 3

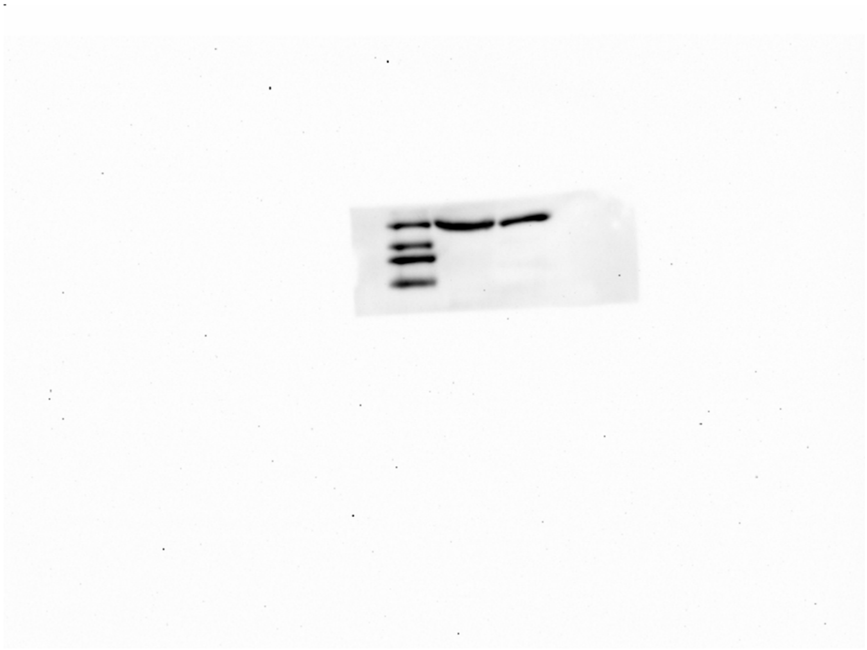

(4) CDK5 1

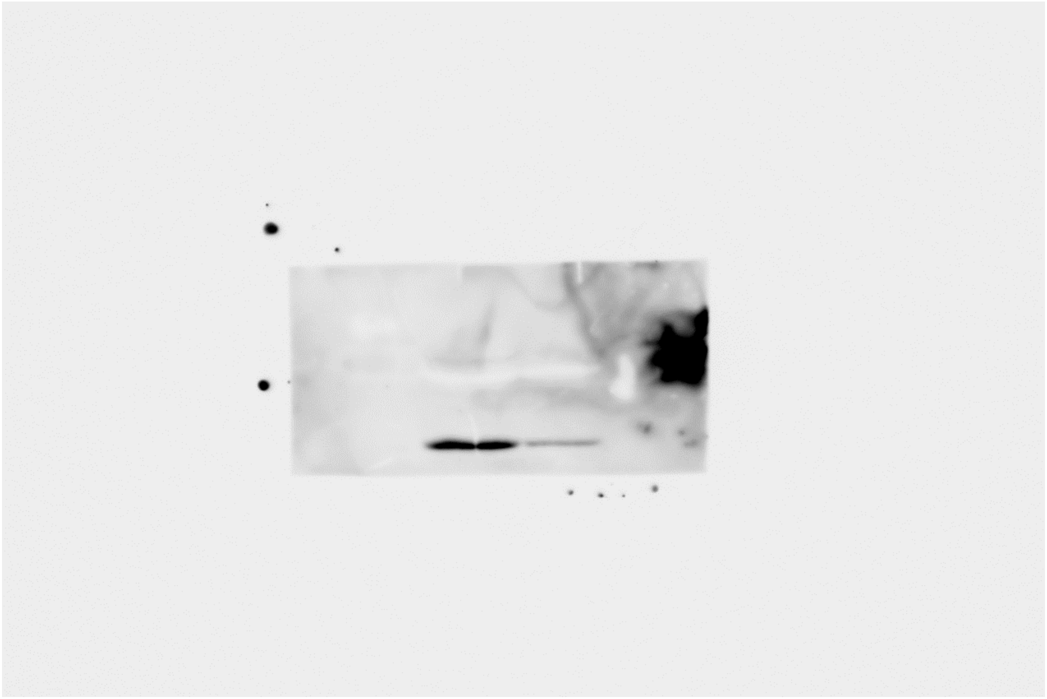

(5) CDK5 2

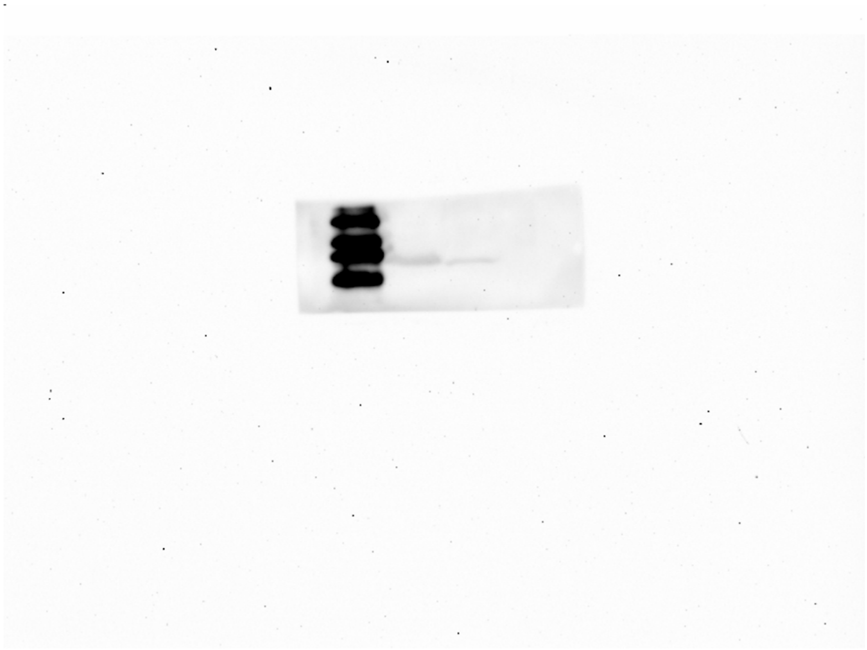

(6) CDK5 3

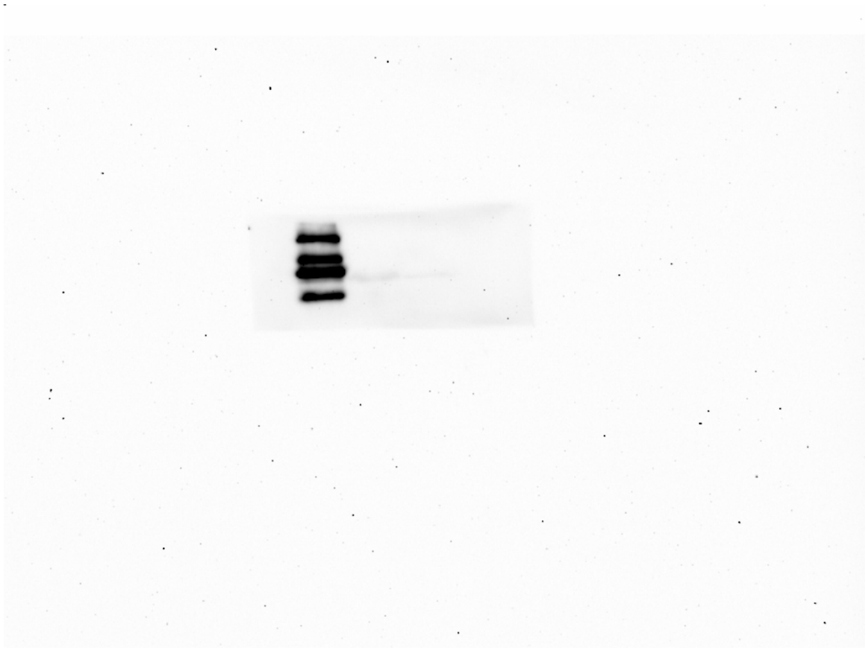

Supplementary Figure S1. Original images of Full Western Blot.
