# Supplementary figures and images for: CDK5/NFAT5-Regulated Transporters Involved in Osmoregulation in Fejervarya cancrivora
Source: Biology (Basel). 2022 Jun 3;11(6):858. doi: 10.3390/biology11060858 (PMC9220195; doi:10.3390/biology11060858)

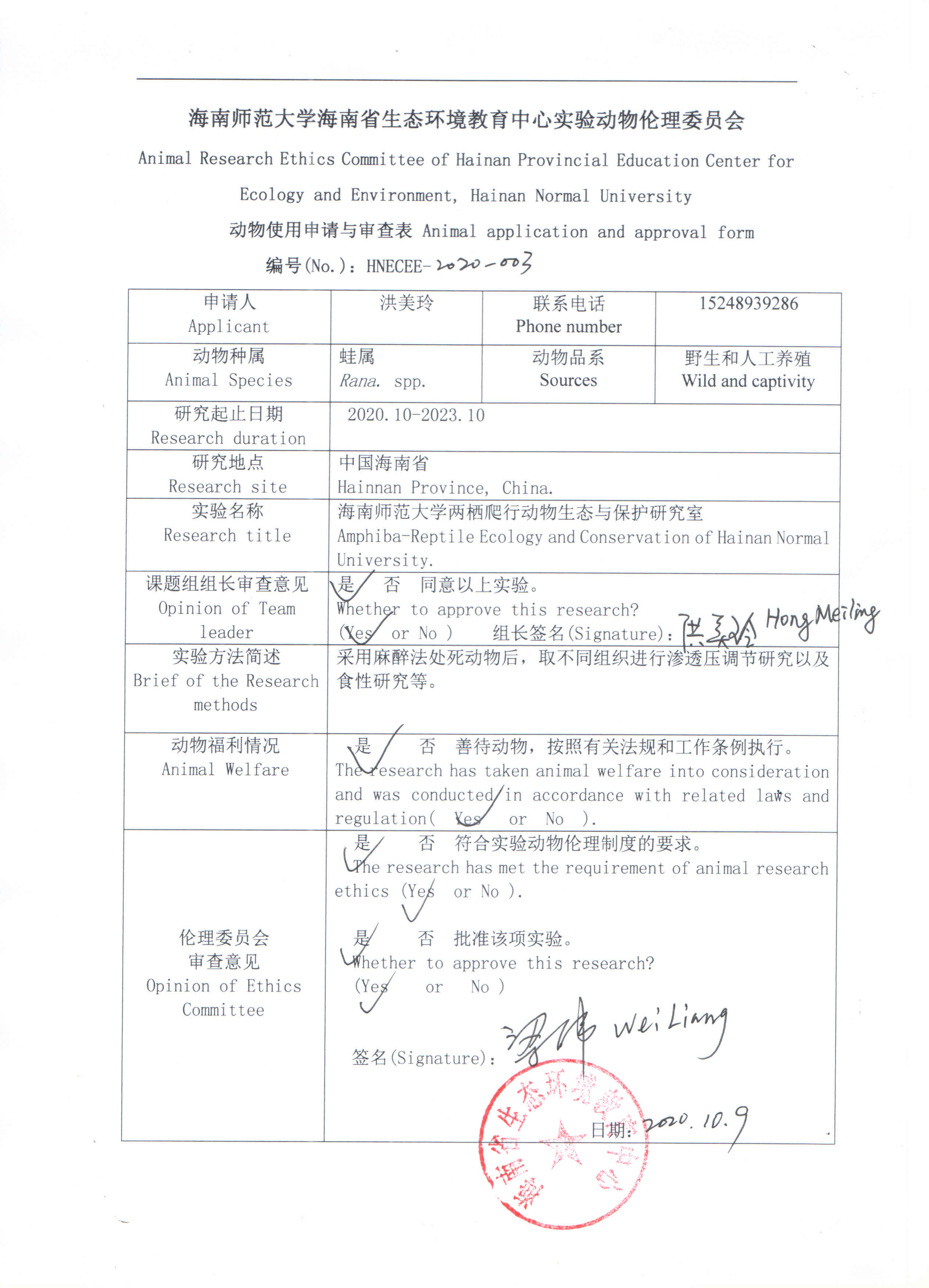

Supplement: Supplementary file 1 [file biology-11-00858-s001.zip › Ethics Number.jpg]
